# Blood demand and utilization among pregnant and postpartum women with and without HIV infections at the university teaching Hospital, Zambia

**DOI:** 10.1038/s41598-025-32500-1

**Published:** 2026-01-16

**Authors:** Udhayashankar Kanagasabai, Michelle S. Chevalier, Clement B. Ndongmo, Bellington Vwalika, Yusuf Ahmed, Dejana Selenic, Anindya K. De, Bakary Drammeh

**Affiliations:** 1 Division of Global HIV and Tuberculosis, Center for Global Health, Centers for Disease Control and Prevention, 1600 Clifton Rd NE, MS E-04, Atlanta, GA 30329 USA; 2 Division of Global HIV and TB, Centers for Disease Control and Prevention, Lusaka, Zambia; 3 University Teaching Hospital Zambia, Lusaka, Zambia; 4 Center for Scientific Review, National Institute of Health, Bethesda, MD USA

**Keywords:** Blood, HIV, Pregnancy, Transfusion, Epidemiology, HIV infections, Haematological diseases

## Abstract

**Background:**

Complications from pregnancy, primarily post-partum hemorrhage, including infections like HIV, are the leading causes of maternal mortality in sub-Saharan Africa (SSA). Blood products are not always available for obstetric emergencies because of chronic shortages in SSA countries. Understanding the quantity of blood products used by pregnant and post-partum women with and without HIV infections could inform strategies for the appropriate use of blood in resource-limited settings.

**Methods:**

A prospective study was conducted of medical charts of all pregnant or postpartum (42 days following delivery) women who presented to the obstetrics ward at the University Teaching Hospital (UTH) Zambia and had blood ordered during their hospital stay from November 2016 to March 2017. Abstracted data from the requisition forms included age, hospital ward, clinical indication for blood transfusion, transfusion history, pregnancy history, blood components requested, number of units requested, and date and time of the request. Data extracted from blood bank records included blood issue date, number of units issued, and pre-transfusion hemoglobin. Variables extracted from the patient’s chart included age, HIV status, pregnancy history, blood component transfused, delivery method, labor complications, and transfusion outcome/adverse events.

**Results:**

A total of 1086 blood requests and charts of women were reviewed in the study at UTH. Blood requests for women living with HIV (WLHIV) and women without HIV infection were 265 (24.4%) and 821 (75.6%), respectively. Of the 1034 blood requests received, WLHIV represented 40.2% (103) of antepartum (*n* = 256) requests and 38.9% (*n* = 303) of postpartum (*n* = 778) blood requests. Of the 43% (465) women who received a transfusion, 47.1% (125) were WLHIV and 41.4% (340) were women without HIV infection. Pregnancy-related indications for transfusion in WLHIV were other 92 (34.7%), postpartum hemorrhage 26 (9.8%), complications of ectopic pregnancy 23 (8.6%), acute anemia 11 (4.1%), placenta abruption 8 (3.0%), uterine rupture 3 (1.1%), disseminated intravascular coagulation (DIC) and HELLP syndrome 1 (0.3%).

**Conclusion:**

HIV infection’s contributory role in blood demand and use among pregnant women in Zambia may be smaller than previously thought. However, findings suggest that continued investments in hemovigilance and appropriate blood use within the clinical setting could improve rational blood use in low-resource settings and maternal outcomes.

## Introduction

The availability of safe blood and blood products has a substantial impact on maternal morbidity and mortality in Sub-Saharan African (SSA) countries, where the burden of HIV is high. In 2017, the maternal mortality ratio (MMR) in low-resource countries was 462 per 100,000 live births versus 11 per 100,000 live births in high-resource countries^1,2^. The leading contributors to the high MMR reported in low-resource countries include post-partum hemorrhage, complications of pregnancy (e.g. Eclampsia, preeclampsia, gestational diabetes), and infectious diseases such as HIV^3^. In Zambia, women (aged 15–49 years) had a higher HIV prevalence 13.9% vs. men 8.0%, according to the 2021 Zambia Population-Based HIV/AIDS Impact Assessment^3^. One study in Zambia shows how HIV prevalence among pregnant women declined from 24.5% in 2003 to 21.4% in 2006^1^. HIV infection in pregnancy has been demonstrated to lead to an 8-fold increase in the risk of maternal death, and studies have shown that women living with HIV (WLHIV) are at increased risk for neonatal and obstetrical complications, including severe anemia^4,5,5–9^. Half of the maternal deaths due to severe bleeding in the world occur in SSA, and about 65% of these deaths occur in the postpartum period^4^. Zambia has invested resources in maternal health, which has seen a reduction in maternal mortality from 398 in 2013–2014 to 278 in 2018^10^. One study in Zambia highlighted hemorrhage as the leading cause of maternal mortality and no positive association with HIV status^10^. The availability and accessibility of safe blood within low-resource settings are key interventions for reducing maternal morbidity and mortality associated with severe bleeding^2,8,11,12^.

For over 30 years, the World Health Organization (WHO) has promoted the adoption of blood safety strategies aimed at ensuring adequate availability and access to safe blood and blood products^5,13,14^. However, despite tremendous strides in increasing total blood collections, the rate of collections in many SSA countries remained below the WHO target of 10 units per 1,000 population per year. Equitable access to blood/blood products and appropriate use remains a challenge in many developing countries^15,16^. Additionally, key characteristics of safe blood transfusion systems, such as patient survival related to blood availability, blood demand (total number of units requested), hemovigilance (monitoring of adverse events), and blood use among pregnant and postpartum women, leave room for improvements. There exists a paucity of literature in SSA on the transfusion process, from the clinicians’ prescription/request for blood, the issuing of blood by the blood bank, transfusion of blood, and monitoring of the transfusion. When funding resources are limited at this critical juncture, a better understanding of the vital role of timely, safe, and adequate blood supply in improving maternal outcomes should be considered a priority.

The Zambia National Blood Transfusion Services (ZNBTS), which operates under the authority of the Zambia Ministry of Health (MOH), was established in 2004 to ensure the efficient and effective implementation of a national blood safety strategy based on five key elements recommended by the World Health Organization (WHO)^17^. While Zambia has documented substantial achievement in reaching WHO blood safety standards, data on quality blood transfusion systems, including patient outcomes following transfusions, is lacking^18,19^.

The overarching aims of this study were: (i) to quantify the blood demand among pregnant and postpartum women living and not living with HIV at a single referral hospital in Zambia, (ii) to assess the use of blood requests, (iii) to describe the prevalence of transfusion-related adverse events among transfusion recipients, and (iv) to describe the maternal outcomes of unmet demand.

## Methods

### Study design and participants

A prospective study was conducted by reviewing medical charts of all pregnant or postpartum (42 days following delivery) women who presented to the obstetrics and gynecological (for early pregnancy complications) wards at the University Teaching Hospital (UTH) Zambia and had blood ordered during their hospital stay from November 2016 to March 2017. All study participants or legal guardians provided informed written consent as per the University Teaching Hospital requirements.

## Sampling and sample size

Power analyses were performed using Power and Sample Size (PASS) version 11.0. The sample size calculations for the proportions’ confidence interval (CI) was based on score tests with continuity correction. To accurately estimate the unmet proportion of blood demand, we set the precision at 0.065 (length of the CI) for the proportion set at 0.5, while factoring in the standard 10% non-response/record loss rate, we calculated that we would need a minimum sample size of 1040 participants. All blood product requests for pregnant and postpartum women in the obstetrics and labor wards at UTH (whole blood/packed red blood cells or blood components (platelets, fresh frozen plasma, or cryoprecipitate) submitted to the blood bank (ZNBTS) were included.

## Study variables

Study variables extracted from the blood request forms included age, sex, clinician indication (reason) for blood transfusion, transfusion history, pregnancy history, blood component requested, number of units requested, date, and time of the request. Data extracted from the blood bank records included blood issue date, number of units issued, and pre-transfusion hemoglobin level. Variables extracted from the patient’s chart included age, HIV status, pregnancy history, blood component transfused, dates and times of blood request and transfusion, delivery method, labor complications, blood loss, transfusion outcome/adverse events, and maternal and neonatal outcomes. For this study, we classified blood requests; as emergency care (immediately), standard [within 12 h], and urgent [within 1 h]. The primary outcomes assessed were blood transfusion requirements, as well as maternal and neonatal outcomes. Other outcomes assessed included transfusion-associated adverse events and demographic and clinical characteristics.

## Data collection

Data were collected from medical charts, lab records, and blood request forms by trained data abstractors using paper abstraction forms that were entered into a Census and Survey Processing System (CSPro) database daily. The database was stored and backed up daily on an encrypted flash drive.

### Statistical analysis

Data analysis was conducted using STATA [*StataCorp. 2017. Stata Statistical Software: Release 15.0. College Station*,* TX: StataCorp LLC*]. Demographic, clinical, and medical data were presented as mean and standard deviation for numeric data, median and interquartile range for skewed quantitative data, and frequency and proportion for categorical data. All variables were summarized on aggregate data stratified by maternal HIV status. T-test/median test and chi-squared test were used to compare various indicators between WLHIV and women without HIV.

### Ethical consideration

This activity was reviewed by the U.S. Centers for Disease Control and Prevention, deemed not research, and was conducted consistent with applicable federal law and CDC policy (45 C.F.R. part 46.102(l)^2^, 21 C.F.R. part 56; 42 U.S.C. Section 241(d); 5 U.S.C. Sect. 552a; 44 U.S.C. Section 3501 et seq). The study was also approved by the Ethics Review Board of the Tropical Diseases Research Centre in Zambia. Personal identifying data were not extracted from medical charts, and all data collected remained anonymous. All data collection forms were stored in locked cabinets and entered into password-encrypted laptops.

## Results

### Baseline characteristics

A total of 1086 women were enrolled in the study at UTH. The majority of women were HIV-negative (*n* = 821, 75.6%) vs. WLHIV (*n* = 265, 24.4%) (Table 1). The median age of WLHIV was 31 years (interquartile range [IQR]: 27–35) vs. women who were HIV-negative was 28 years (IRQ: 23–33) (*p* < 0.01). The median hemoglobin level at first transfusion between both groups: 7.6 g/dl (IQR: 5.4–10.0) for WLHIV and 8 g/dl (6.1–11.0) for those HIV-negative. Among WLHIV, 207 (78.1%) were on antiretroviral therapy. The major pregnancy type was singleton (WLHIV: *n* = 156, 81.7%; HIV-negative: *n* = 485, 84.9%), followed by twins (WLHIV: *n* = 7, 3.7%; HIV-negative: *n* = 35, 6.1%). The primary methods of delivery included vaginal delivery (WLHIV: *n* = 68, 25.7%; HIV-negative: *n* = 485, 32.3%; *p* = 0.04), cesarean delivery (WLHIV: *n* = 73, 27.6%; HIV-negative: *n* = 233, 28.4%, surgical removal of ectopic pregnancy (WLHIV: *n* = 25, 9.4%; HIV-negative: *n* = 45, 5.5%; *p* = 0.03), medical termination (WLHIV: *n* = 44, 16.6%; HIV-negative: *n* = 145, 17.4%), and other methods (WLHIV: *n* = 13, 4.9%; HIV-negative: *n* = 10, 1.2%; *p* < 0.01). The average blood loss per delivery was estimated as 400 ml in WLHIV (IQR: 200–675) and 400 ml (IQR: 200–750) for those HIV-negative. The top three co-morbid conditions were tuberculosis (WLHIV: *n* = 15, 5.7%; HIV-negative: *n* = 2, 0.2%; *p* < 0.01), chronic anemia (WLHIV: *n* = 5, 1. 9%; HIV-negative: *n* = 5, 0.6%; *p* = 0.07), and diabetes (WLHIV: *n* = 3, 1.1%; HIV-negative: *n* = 1, 0.1%; *p* = 0.05).

**Table 1:** Baseline characteristics of study participants stratified by HIV status at UTH, Lusaka, Zambia 2016 (N = 1086).

Maternal characteristics	Maternal HIV status		*P* Value
	HIV + (n = 265)	HIV- (n = 821)	
Age (yrs) (Median, IQR)	31 (27–35)	28 (23–33)	** < 0.01**
Hb at time of 1st transfusion (g/dl) (median, IQR)	8 (5.4–10.0)	8 (6.1–11.0)	0.44
No. of women on ART (n, %)	207 (78.1%)	3 (0.4%)	** < 0.01**
Labor characteristics			
Gestational age at admission (wks) (median, IQR)	34 (24–38)	36 (30–38)	** < 0.01**
Total live births (Median, IQR)	3 (2–4)	2 (1–4)	0.01
Gravidity (Median, IQR)	4 (2–5)	3 (2–4)	** < 0.01**
Pregnancy type (N, %)			
Singleton	156 (81.8)	485 (84.9)	0.95
Twins	7 (3.7)	35 (6.1)	0.23
Multiples (3 + fetuses)	1 (0.5)	6 (1.1)	0.53
Other	27 (14.1)	45 (5.5)	**0.01**
Delivery method			
Vaginal delivery (vaginal, vacuum, or forceps delivery)	68 (25.7)	265 (32.3)	**0.04**
Cesarean delivery	73 (27.6)	233 (28.4)	0.79
Surgical removal of ectopic pregnancy	25 (9.4)	45 (5.5)	**0.03**
Medical termination	44 (16.6)	145 (17.4)	0.78
Other methods	13 (4.9)	10 (1.2)	** < 0.01**
Total estimated blood loss (ml) (Median, IQR)	400 (200–675)	400 (200–750)	0.34
Co- Morbid conditions			
Tuberculosis	15 (5.7)	2 (0.2)	** < 0.01**
Heart disease	1 (0.4)	3 (0.4)	1
Chronic high blood pressure	2 (0.8)	24 (2.9)	0.06
Renal disease	2 (0.8)	4 (0.5)	0.64
Diabetes	3 (1.1)	1 (0.1)	**0.05**
Chronic anemia	5 (1.9)	5 (0.6)	0.07
Asthma	0 (0)	0 (0)	⁻
Malaria in current pregnancy	1 (0.4)	5 (0.6)	1
STI‡	0 (0)	3 (0.4)	1
Epilepsy	0 (0)	1 (0.1)	1
Sickle cell anemia	1 (0.4)	3 (0.4)	1
Other	8 (3.0)	13 (1.6)	0.2

HIV, Human immunodeficiency virus; IQR, Interquartile range, UTH, University teaching hospital

Significant values are in bold

### Clinical indications for request of blood products

The number of pregnancy-related indications among WLHIV was 164, and similar to those of HIV-negative women (489) (Table 2). None of these conditions, except for acute anemia, was statistically significant among WLHIV compared to women who were HIV-negative. The pregnancy-related indications were: postpartum hemorrhage (WLHIV: *n* = 26, 15.9%; HIV-negative: *n* = 119, 24.3%), complications of ectopic pregnancy ( WLHIV: *n* = 23, 14%; HIV negative: *n* = 44, 9%), acute anemia (WLHIV: *n* = 11, 6.7%; HIV-negative: *n* = 13, 3.7%; *p* = 0.03), placental abruption (WLHIV: *n* = 8, 4.9%; HIV-negative: *n* = 38, 7.8%),, disseminated intravascular coagulation (DIC) and hemolysis, elevated liver enzymes, low platelet count (HELLP) syndrome (WLHIV: *n* = 1, 0.6%; HIV-negative: *n* = 11, 2.2%), uterine rupture (WLHIV: *n* = 3, 1.8%; HIV-negative: *n* = 9, 1.8%), and other (WLHIV: *n* = 92, 56.1%; HIV-negative: *n* = 255, 52.1%) (Table 2).

**Table 2:** Medical Indications for transfusion among study participants at UTH in Lusaka, Zambia (N = 1086)*

	Maternal HIV status		*P* Value
	HIV + (n = 265)	HIV- (n = 821)	
**Medical indications for blood or component transfusion** ^±^			
Pregnancy-related causes	**164**	**489**	**0.52**
Placenta abruption	8 (4.9)	38 (7.8)	0.30
Postpartum hemorrhage (PPH)	26 (15.9)	119 (24.3)	0.06
Disseminated Intravascular Coagulation (DIC) and HELLP syndrome*	1 (0.6)	11 (2.2)	0.31
Uterine rupture	3 (1.8)	9 (1.8)	1
Gestational thrombocytopenia	0 (0)	0 (0)	⁻
Complications of ectopic pregnancy	23 (14)	44 (9)	0.06
Acute anemia (non-related to PPH)	11 (6.7)	13 (2.7)	**0.03**
Other	92 (56.1)	255 (52.1)	0.29
Non-pregnancy related causes	**7**	**17**	**0.63**
Motor-vehicle accident	1 (14.3)	7 (41.2)	0.69
Massive trauma	0 (0)	1 (5.9)	1
Idiopathic thrombocytopenic purpura	0 (0)	0 (0)	⁻
Complications of malaria	0 (0)	1 (5.9)	1
Sickle cell anemia (SCA)	0 (0)	0 (0)	⁻
Chronic anemia/Anemia of chronic disease (excluding SCA)	1 (0.4)	0 (0)	0.24
Other	**5** **(71.4)**	**8** **(47.1)**	**0.33**

HELLP,  Hemolysis elevated liver enzymes and low platelet count; HIV,  Human immunodeficiency virus; IQR, Interquartile range; UTH, University teaching hospital

Significant values are in bold

*Out of 1186 records HIV status were missing for 100 and excluded from the analysis

±Includes only indications that were documented in the charts

### Transfusion-associated adverse events

There were two (0.7%) WLHIV and six (0.6%) HIV-negative women without documentation of immediate transfusion adverse events (Table 3). Acute hemolytic reactions and transfusion-related lung injury were not reported, whereas fever less than 100.4 F and allergic reactions were only reported among those HIV-negative.

**Table 3:** Transfusion-associated adverse events, and maternal and neonatal outcomes among study recipients at UTH in Lusaka, Zambia, 2016 (N = 1086).

Maternal characteristics	Maternal HIV status		*P* Value
	HIV + (n = 265)	HIV- (n = 821)	
**Immediate transfusion adverse events**			
Yes	2 (.7)	6 (.6)	0.68
No	158 (59.6)	511 (62.2)	0.47
Type of immediate transfusion adverse event			
Fever (> 100.4 F)	0 (0)	2 (0.2)	1
Allergic reaction (mild or severe)	0 (0)	2 (0.2)	1
Acute hemolytic reaction	0 (0)	0 (0)	–
Transfusion-related acute lung injury (TRALI)	0 (0)	0 (0)	–
Other	2 (0.7)	2 (0.2)	0.25
Labor complications			
Placental abruption	2 (0.8)	12 (1.5)	0.54
Postpartum hemorrhage (PPH)	29 (10.9)	134 (16.3)	**0.04**
Obstructed labor	0 (0)	1 (0.1)	1
Hypertensive disorders (DIC, HELLP syndrome, pre-eclampsia, eclampsia)	8 (3.0)	41 (5.0)	0.23
Uterine rupture	1 (0.4)	7 (0.9)	0.69
Septic abortion	4 (1.5)	7 (0.9)	0.48
Anemia	33 (12.5)	83 (10.1)	0.28
Other	17 (6.4)	65 (7.9)	0.5
Maternal mortality			
Yes	7 (2.6)	13 (1.6)	0.29
No	258 (97.4)	808 (98.4)	
Neonatal outcomes (median, IQR)			
1-min APGAR score	9 (3.5–9)	8 (5–9)	0.82
5-min APGAR score	9 (4–9)	9 (7–9)	0.93
Fetal weight at birth (g)	2600 (1900–3000)	2800 (2200–3300)	0.02
Neonatal mortality/stillbirth			
Yes	35 (13.2)	115 (14.0)	0.84
No	230 (86.8)	706 (86.0)	

HIV, Human immunodeficiency virus; HELLP, Hemolysis, elevated liver enzymes, and low platelet count; IQR, Interquartile range, UTH, University teaching hospital

Significant values are in bold

*Out of 1186 records HIV status were missing for 100 and excluded from the analysis

### Maternal and neonatal outcomes among transfused patients

The most common complications of labor for WLHIV and HIV-negative women were, respectively: postpartum hemorrhage (*n* = 29, 10.9% vs. *n* = 134, 16.3%; *p* = 0.04); anemia (*n* = 33, 12.5% vs. *n* = 83, 10.1%); hypertensive disorders (*n* = 8, 3.0% vs. *n* = 41,5.0%); septic abortion (*n* = 4, 1.5% vs. *n* = 7, 0.9%); and placental abruption (*n* = 2, 0.8% vs. *n* = 12, 1.5%) (Table 3). Among all labor complications, postpartum hemorrhage was statistically significant among WLHIV compared to HIV-negative women. Maternal mortality was reported among seven (2.6%) WLHIV and 13 (1.6%) HIV-negative women. Among neonates born to WLHIV, the median one-minute APGAR score was nine (IQR: 3.5-9) vs. eight (IQR: 5–9) among HIV-negative women. The median five-minute APGAR score of nine was the same for both groups (WLHIV range: 4–9, HIV-negative range: 7–9). Neonatal mortality was 35 (13.2%) among WLHIV women and 115 (14.0%) among HIV-negative women.

### Clinical blood demand and unmet need

During the study period, there were a total of 1034 requests for blood/blood components with 256 (96.6%) for WLHIV and 778 (94.8%) for HIV-negative women (Table 4). Among both WLHIV and HIV-negative women admitted during the study, the median number of units requested was four (IQR: 3–5). The major period for blood requests among WLHIV were antepartum (*n* = 103, 40.2%) and postpartum (*n* = 153,59.8%), and, among HIV-negative women, antepartum (*n* = 303, 38.9%) and postpartum (*n* = 475,61.0%). The most common type of request for blood and blood components was for emergency care (*n* = 335), followed by standard [within 12 h] (*n* = 425) and urgent [within 1 h] (*n* = 314) care (Table 4). The median number of whole blood units issued was 2 (IQR: 1–1) for WLHIV and 2 (IQR: 1–2) for HIV-negative women. The total number of transfusions among WLHIV was 125 (47.2%) vs. 340 (41.4%) for HIV-negative women.

**Table 4:** Clinical blood demand and unmet need among study participants at UTH, Lusaka Zambia, 2016  (N = 1086).

Maternal characteristics	Maternal HIV status		*P* Value
	HIV + (n = 265)	HIV- (n = 821)	
Median number of units requested (Median, IQR)	4 [3–5]	4 [3–5]	0.9
Blood Bank requests			
Total Number of requests for blood/components	256 (96.6)	778 (94.8)	0.25
Antepartum	103 (40.2)	303 (38.9)	0.61
Postpartum	153 (59.8)	475 (61.1)	0.94
Urgency level of blood request			
Emergency	89(34.9)	246 (30.9)	0.29
Urgent	93(24.7)	221 (27.8)	0.53
Standard	101(39.6)	324 (40.8)	0.72
Number of blood units requested (median, IQR)	4.4(3.3)	4.7 (3.8)	0.9
Whole blood (WB) and packed red blood cells (PRBC)	4 (3–4)	4 (3–4)	
Components (platelets, fresh frozen plasma, and cryoprecipitate)	0 (0–0)	0 (0–0)	
Time for fulfillment of blood request (Median, IQR) (days)			
Whole blood	0 (0–1)	0 (0–1)	0.31
Packed red cells	1 (0–1)	0 (0–1)	0.60
Components (platelets, fresh frozen plasma, and cryoprecipitate)	0 (0–1)	0 (0–1)	0.71
Number of units issued (median, IQR)	1 (1–2)	1 (1–2)	0.14
Whole blood (WB) and Packed Red Blood Cells (PRBC)	2 (1–1)	2 (1–2)	0.32
Components (platelets, fresh frozen plasma, and cryoprecipitate)	4 (4–4)	4 (2–4)	0.53
Transfusions			
Total number of patients transfused	125 (47.2)	340 (41.4)	0.1
Total number of patients not transfused	140 (52.8)	481 (58.6)	0.1
Number of blood units transfused (median, IQR)	2 (1–2)	2 (1–2)	0.67
Whole blood (WB) and packed red blood cells (PRBC)	2 (1–2)	2 (1–2)	0.95
Components (platelets, fresh frozen plasma, and cryoprecipitate)	4 (2–4)	4 (2–4)	0.99
Reasons for non-receipt of blood and/or components			
Blood or component not available	40 (15.1)	102 (12.4)	0.26
No longer medically indicated	87 (32.8)	282 (34.4)	0.65
Patient expired /died	1 (0.4)	4 (0.5)	1
Patient refused	5 (1.9)	16 (2.0)	1
Delay in blood processing	33 (12.5)	48 (5.9)	** < 0.01**
Other	2 (0.8)	8 (1.0)	1

HIV, Human immunodeficiency virus; IQR, Interquartile range, UTH, University teaching hospital

Significant values are in bold

*Out of 1186 records HIV status were missing for 100 and excluded from the analysis

The reasons for non-receipt of blood among WLHIV women were blood/component was not available (*n* = 40, 15.1%) vs. HIV-negative women (*n* = 102; 12.5%), no longer medically indicated for WLHIV (*n* = 87; 32.8%) for vs. (*n* = 282; 34.4%) HIV-negative, delay in processing blood for WLHIV(*n* = 33; 12.5%) vs. (*n* = 48; 5.9%) HIV-negative, and the patient refused transfusion for WLHIV(*n* = 5; 1.9%) vs. (*n* = 16; 2.0%) HIV-negative (Table 4).

The greatest number of units ordered were made by the postnatal/antenatal (*n* = 597, 56.2%) and gynecological (*n* = 322, 28.2%) wards (Table 5).

**Table 5:** Clinical blood demand and use by maternity wards at UTH, Lusaka Zambia, 2016 (N = 1086).

Units ordered	Frequency	Labor & delivery ward	Special observations unit	Antenatal/ postnatal ward	Emergency gynecology ward	Gynecology ward
	*n*	*n (%)*	*n(%)*	*n(%)*	*n(%)*	*n(%)*
Blood units ordered by hospital wards where pregnant and post-partum women were admitted^±^						
0	29	0 (0)	0 (0)	14 (48.3)	4 (13.8)	11 (37.9)
1	85	1 (1.2)	5 (5.8)	28 (32.6)	11 (12.8)	40 (46.5)
2	148	1 (0.7)	1 (0.7)	89 (60.1)	16 (10.8)	41 (27.7)
3	156	1 (0.6)	1 (0.6)	105 (67.3)	12 (7.7)	37 (23.7)
4	413	7 (1.7)	12 (2.9)	267 (64.7)	32 (7.8)	95 (23.0)
5	132	3 (2.3)	2 (1.5)	53 (40.2)	19 (14.4)	55 (41.7)
6	56	5 (8.9)	2 (3.6)	30 (53.6)	5 (8.9)	14 (25)
7	20	0 (0)	2 (10.0)	10 (50.0)	0 (0)	8 (40)
8	27	3 (11.1)	1 (3.7)	15 (55.6)	1 (3.7)	7 (25.9)
9	10	1 (10.0)	0 (0)	5 (50.0)	1 (10.0)	2 (20.0)
10	27	1 (3.7)	3 (11.1)	12 (44.4)	0 (0)	11 (40.7)
11	10	3 (30)	1 (10.0)	5 (50.0)	1 (10.0)	0 (0)
12	10	0 (0)	2 (20.0)	4 (4.00)	0 (0)	4 (40.7)
13	15	1 (6.7)	5 (33.3)	9 (60.0)	0 (0)	0 (0)
14	15	1 (6.7)	3 (20.0)	7 (46.7)	0 (0)	4 (26.7)
15 +	26	3 (11.1)	8 (29.6)	11 (40.7)	0 (0)	4 (14.8)
Total	**1179**	**31** **(2.6)**	**48 (4.1)**	**664 (56.4)**	**102 (8.7)**	**333 (28.2)**
Blood units issued by blood bank from units ordered						
0	674	7 (1.0)	17 (2.5)	453 (67.2)	51 (7.6)	146 (21.7)
1	179	2 (1.1)	4 (2.2)	71 (39.2)	21 (11.6)	81 (44.8)
2	210	11 (5.2)	13 (6.2)	89 (42.4)	22 (10.5)	75 (35.7)
3	45	2 (4.4)	2 (4.4)	20 (44.4)	4 (8.9)	17 (37.8)
4	36	2 (5.6)	4 (11.1)	17 (47.2)	3 (8.3)	10 (27.8)
5	6	1 (16.7)	2 (33.3)	3 (50.0)	0 (0)	0 (0)
6	11	1 (9.1)	2 (18.1)	6 (54.6)	1 (9.0)	1 (9.0)
7	6	3 (50.0)	1 (16.7)	1 (16.7)	0 (0)	1 (16.7)
8 +	11	2 (16.7)	3 (25.0)	4 (33.3)	0 (0)	2 (16.7)
Total	**1178**	**31 (2.6)**	**48 (4.1)**	**664 (56.4)**	**102 (8.7)**	**333 (28.3)**
Blood units transfused from units issued by hospital wards						
0	665	7 (1.1)	19 (2.9)	452 (68.0)	52 (7.8)	135 (20.3)
1	183	2 (1.1)	4 (2.2)	66 (35.7)	20 (10.8)	91 (49.2)
2	210	11 (5.2)	12 (5.7)	93 (44.3)	21 (10)	73 (34.8)
3	51	4 (7.8)	2 (3.9)	18 (35.3)	5 (9.8)	22 (43.1)
4	34	3 (8.8)	4 (11.8)	17 (50.0)	3 (8.8)	7 (20.6)
5	9	1 (11.1)	4 (44.4)	2 (22.2)	0 (0)	2 (22.2)
6	10	1 (10.0)	0 (0)	7 (70.0)	1 (10.0)	1 (10.0)
7	7	1 (14.3)	2 (28.6)	4 (57.1)	0 (0)	0 (0)
8	9	1 (10.0)	1 (10.0)	5 (50.0)	0 (0)	2 (20.0)
Total	**1178**	**31 (2.6)**	**48 (4.1)**	**664 (56.4)**	**102 (8.7)**	**333 (28.3)**

HIV, Human immunodeficiency virus; IQR, Interquartile range, UTH, University teaching hospital

Significant values are in bold

± 3 units that did not belong to one of the wards above were excluded

*Out of 1186 records HIV status were missing for 100 and excluded from the analysis

The most frequent number of blood units issued during the study period was two units (*n* = 89, 42.4%) for the postnatal/antenatal ward and one unit (*n* = 81, 44.8%) by the gynecological ward. The largest number of transfusions were done in the postnatal/antenatal (mode = 2 units, *n* = 93, 44.2%) and gynecological (mode = 1 unit, *n* = 91; 49.1%) wards. A total of 1152 requests for blood were made by all wards, 507 orders were issued, and 516 orders were transfused (Table 5).

## Discussion

The availability of safe and adequate access to blood transfusions is a cornerstone in the fight to reduce maternal mortality, especially among WLHIV. Our findings did not show that HIV infection led to a higher proportion of women receiving a transfusion or an increase in maternal mortality (Table 3). However, we did find a mismatch between the number of transfusion requests made by clinicians versus what was issued by the blood bank and what was transfused to patients.

Our study sheds light on whether HIV infection increases the demand and use of blood or blood products in the clinical setting. This problem is often overlooked in clinical literature concerning HIV-related maternal mortality in low-resource settings. In many settings, WLHIV may be at increased risk of malaria complications, including severe anemia and obstetric hemorrhage, all of which increase her risk of requiring blood or blood products during labor and delivery and, subsequently, increased risk of maternal morbidity and mortality^1,6,7,11,19,20^. Recent modeling analysis has tried to capture the impact of HIV-related indirect maternal deaths, showing that for countries with a generalized HIV epidemic and high HIV prevalence, HIV/AIDS is a leading cause of death during pregnancy and post-delivery^11,21^. In 2017, it was estimated that 3,600 HIV-related indirect maternal deaths occurred worldwide^2^. While one of our primary aims was to understand blood demand among this population, our study did not show that there was a significant difference in blood demand and outcomes between WHIV and HIV-negative women. The lack of difference between the two groups could be attributed to the improved uptake and accessibility of ART, as well as viral suppression, among WLHIV, which has occurred in recent years as access to HIV treatment has increased. Another hypothesis is that WLHIV is recommended to have frequent clinic visits, increasing the likelihood of an early diagnosis of complications and comorbidities such as anemia during pregnancy^22–26^.

The use of blood and the request for blood is primarily driven by the obstetric and surgical needs of patients in a hospital. Several regional studies (Tanzania, Kenya, and Uganda) highlighted the critical role blood availability plays in reducing maternal mortality^24,27,28^. Our findings help our understanding of the primary clinical indications for blood use among pregnant women, including those living with HIV. Hemorrhage remains the leading cause of maternal death worldwide, representing 27% of maternal deaths, of which more than two-thirds are attributed to postpartum hemorrhage^29^. A high maternal mortality rate is usually related to a high demand for blood in hospital settings, especially in low-resource settings^18,20,29,30^. We found a significant difference between study populations in postpartum hemorrhage (PPH) rates. A similar study conducted in Tanzania saw that the most common indications among all pregnant women for blood transfusions at hospitals were malaria (31.4%) and maternal hemorrhage (17.4%)^26^. The use and availability of blood for pregnant women are also dependent on the location of the health facility, with increased availability seen in urban settings versus rural ones^24^. While other reasons for blood transfusions during pregnancy can occur, these are uncommon and were often not captured within the hospital charts and available for abstraction. The low maternal mortality seen in our study could be attributed to the overall improved maternal outcomes, the decline in HIV prevalence, increased access to ART, and improved viral suppression of WLHIV in Zambia and globally^2,11,27,31–35^. The high neonatal mortality rate (13.2%) among WLHIV women and 14.0% among HIV-negative women) presented in this study are similar to those observed in other studies conducted in Zambia^2^. Other clinical factors that can influence the use of blood in a hospital setting for women include the frequency of cesareans performed and anemia. Interestingly, our findings did not show a significant difference in the number of cesarean deliveries performed among WLHIV compared to other study participants. These findings suggest that pre-labor elective cesareans were not common among WLHIV and possible adherence to recent WHO recommendations for low-resource settings^11,26,27,31,33^. No significant associations were seen between anemia patients and the need for transfusions. However, the high number of participants with missing hemoglobin levels in their medical records highlights the need for documentation and improved record-keeping.

Hemovigilance is the monitoring of the entire transfusion procedure, from the point of blood collection to the follow-up of recipients of blood donations^3^. Many African countries do not have effective quality systems that include hemovigilance^13,36^. Despite some literature indicating that transfusion incidents are numerous and frequent, there is scarce documentation as to the exact frequency of occurrence within the setting of low-income countries^20^. The more common and immediate adverse reactions of fever and allergy reactions were seen in our study and are similar to those seen in other settings^20,37^. Our study found that a few transfusion-related adverse events could be due to poor documentation practices or a poorly functioning hemovigilance system. Additionally, blood banks could work with healthcare worker organizations to increase knowledge and awareness of what adverse reactions are related to blood transfusions and how best to document this. These findings highlight the importance of improved blood transfusion systems, which include hemovigilance as part of a broader blood safety approach^37–39^.

We observed a significant mismatch between the number of blood requests generated from the wards, the number of units of blood issued by the blood bank, and the actual number of units transfused. The number of requests made for blood outnumbered the number of blood orders filled in the study setting. This may be a unique finding to UTH, given that it is a teaching and referral hospital and, as such, has procedures and protocols in place that encourage ordering blood for all pregnant women who may be at risk of hemorrhage at admission. Additionally, the country has protocols and recommendations to ensure that surgeons conservatively order blood even when it is not clinically indicated (e.g., standby blood for elective cesarean section). However, a study in Tanzania found that tertiary-level hospitals had the highest percentage (32.0%) of inappropriate blood requests, followed by primary-level (16.4%) and secondary-level hospitals (15.1%)^39^. These findings emphasize the need for clear hospital protocols and national policies that determine the use of blood and blood products. The mismatch between blood requests and filled blood orders implies an unmet demand for blood at the study site. However, due to poor documentation, it was difficult to ascertain if the unmet demand for blood, which was primarily due to blood being unavailable or delayed, had an untold impact on maternal outcomes. The few maternal deaths documented during the study were not directly linked to blood shortages. Furthermore, these findings substantiate the assumptions that there may be an unmet demand for blood that often goes unnoticed. Overall, there is a lack of maternal mortality data from rural and urban healthcare facilities in SSA that link obstetric hemorrhage to a shortage of blood supply^29,40–42^. Given the small sample size used and the study’s location at a teaching hospital with higher resources, the relationship between maternal mortality and unmet blood demand could not be fully explored. Further research that includes rural and urban health centers would better help quantify this mismatch and unmet demand.

Zambia could benefit from adapting successful policies implemented in the region. Nigeria has implemented training of healthcare workers and midwives on the appropriate use of blood in managing maternal hemorrhage^43,44^. Malawi has implemented mobile blood donation units that travel to remote areas to encourage donations and deliver blood supplies to rural health facilities^12^. While Ghana and several PEPFAR-supported countries have leveraged Global Fund and other international funding streams to build and strengthen their blood transfusion systems^14,45^.

This study is subject to several limitations. First, data for several variables were missing, which might have led to lower power levels in comparisons. For example, the hemoglobin level at first transfusion was unknown in half of the study participants, making it challenging to conclude anemia. Second, the lack of a hemovigilance system and training of clinicians on the appropriate use of blood may have led to the under-reporting of adverse events related to blood transfusions. Third, the study did not collect information on whether WLHIV was virally suppressed. As such, investigators were unable to determine if the low maternal mortality among WLHIV was impacted by these unaccounted variables. Finally, the algorithm used for clinical demand, appropriate use of blood, and unmet needs may not have accounted for all considerations made by the attending clinician.

## Conclusions

In summary, HIV infection may have an indirect contributory role to blood demand needs for pregnant women in this teaching hospital in Zambia. However, further research in other settings is required. Additional research is needed to understand the relationship between frequent clinic visits recommended for WLHIV and maternal outcomes, compared to those in the general population. Despite many improvements in safe and adequate blood availability, much remains to be done to strengthen hemovigilance and appropriate use of blood in SSA. Health facilities and countries should ensure that explicit policies delineate the proper clinical use of blood and the establishment of hemovigilance systems. Further clinical studies on the appropriate use and demand for blood in clinical settings in SSA are critical for understanding how to meet the need for safe and quality blood.

## Data Availability

The data sets generated during the study are available from the corresponding author on reasonable request.
